# B-Lymphocyte Phenotype Determines T-Lymphocyte Subset Differentiation in Autoimmune Diabetes

**DOI:** 10.3389/fimmu.2019.01732

**Published:** 2019-07-25

**Authors:** Leire Egia-Mendikute, Berta Arpa, Estela Rosell-Mases, Marta Corral-Pujol, Jorge Carrascal, Jorge Carrillo, Conchi Mora, Harold Chapman, Anaïs Panosa, Marta Vives-Pi, Thomas Stratmann, David Serreze, Joan Verdaguer

**Affiliations:** ^1^Immunology Unit, Department of Experimental Medicine, Faculty of Medicine, IRBLleida, University of Lleida, Lleida, Spain; ^2^The Jackson Laboratory, Bar Harbor, ME, United States; ^3^Microscopy and Flow Cytometry Facility, IRBLleida, Universitat de Lleida, Lleida, Spain; ^4^Immunology Section, Germans Trias i Pujol Research Institute, Autonomous University of Barcelona, Barcelona, Spain; ^5^CIBER of Diabetes and Associated Metabolic Diseases, Instituto de Salud Carlos III, Madrid, Spain; ^6^Department of Cell Biology, Physiology and Immunology, Faculty of Biology, University of Barcelona, Barcelona, Spain

**Keywords:** type 1 diabetes, NOD mouse, transgenic mouse model, B-lymphocyte phernotype, T-lymphocyte phernotype

## Abstract

Previous studies indicate that B-lymphocytes play a key role activating diabetogenic T-lymphocytes during the development of autoimmune diabetes. Recently, two transgenic NOD mouse models were generated: the NOD-*PerIg* and the 116C-NOD mice. In NOD-*PerIg* mice, B-lymphocytes acquire an activated proliferative phenotype and support accelerated autoimmune diabetes development. In contrast, in 116C-NOD mice, B-lymphocytes display an anergic-like phenotype delaying autoimmune diabetes onset and decreasing disease incidence. The present study further evaluates the T- and B-lymphocyte phenotype in both models. In islet-infiltrating B-lymphocytes (IIBLs) from 116C-NOD mice, the expression of H2-K^d^ and H2-A^g7^ is decreased, whereas that of BAFF, BAFF-R, and TACI is increased. In contrast, IIBLs from NOD-*PerIg* show an increase in CD86 and FAS expression. In addition, islet-infiltrating T-lymphocytes (IITLs) from NOD-*PerIg* mice exhibit an increase in PD-1 expression. Moreover, proliferation assays indicate a high capacity of B-lymphocytes from NOD-*PerIg* mice to secrete high amounts of cytokines and induce T-lymphocyte activation compared to 116C B-lymphocytes. This functional variability between 116C and *PerIg* B-lymphocytes ultimately results in differences in the ability to shape T-lymphocyte phenotype. These results support the role of B-lymphocytes as key regulators of T-lymphocytes in autoimmune diabetes and provide essential information on the phenotypic characteristics of the T- and B-lymphocytes involved in the autoimmune response in autoimmune diabetes.

## Introduction

CD4+ and CD8+ T-lymphocytes are considered to be the major effectors of ß-cell damage during the development of autoimmune diabetes in both NOD mice and humans. However, T-lymphocytes require the intervention of antigen presenting cells (APCs), such as B-lymphocytes, which drive and modulate their response. The involvement of B-lymphocytes in autoimmune diabetes development was proved when disease protection was observed in NOD mice after introducing the *Ig*μ^*null*^ mutation or after anti-B lymphocyte antibody treatments (1–8). Further studies indicated that B-lymphocytes promote autoimmune diabetes development through their APC function activating ß-cell-reactive cytotoxic T-lymphocytes (CTLs) (9, 10). In parallel to this diabetogenic role, a protective effect of some B-lymphocytes on autoimmunity through a potential regulator/suppressor mechanism has been described. Adoptive transfer of LPS-activated splenic B-lymphocytes protects prediabetic NOD female mice from disease development by triggering the apoptosis of ß-cell-reactive CTLs or/and inhibiting APC activity (11).

Two transgenic NOD mouse models, the 116C-NOD and the NOD-*PerIg* mice (12, 13), have been recently generated to analyze the natural B-lymphocyte immune response during autoimmune diabetes development. On the one hand, the 116C-NOD mouse model expresses the Ig genes from an islet-infiltrating B lymphocyte of a diabetes resistant but insulitis prone (8.3-NODxNOR)F1 mouse with pancreatic islet ß-cell specificity (14). Autoimmune diabetes incidence is decreased in 116C-NOD mice compared to NOD mice (Supplementary Figure 1). B-lymphocytes from 116C-NOD mice display an anergic-like phenotype but retain the ability to express some co-stimulatory molecules after activation and induce a T cell shift toward a Th17 phenotype. On the other hand, the NOD-*PerIg* mouse expresses a representative Ig of a large proportion of naturally occurring islet-infiltrating B-lymphocytes in NOD mice recognizing the neuronal antigen peripherin (14, 15). In contrast to 116C-NOD, B-lymphocytes from NOD-*PerIg* mice acquire an activated proliferative phenotype and promote an accelerated autoimmune diabetes development (Supplementary Figure 1). Despite the differences observed in autoimmune diabetes development, B-lymphocytes from 116C-NOD and NOD-*PerIg* still share certain phenotypic and functional similarities, such as an enlargement of the marginal zone B-lymphocyte subset, and stimulation-induced cytokine production. Thus, the present study aims at studying in depth the functional and phenotypic differences between T- and B-lymphocytes in both mouse models, to better understand the mechanisms underlying the function of both cell populations in inhibiting or accelerating autoimmune diabetes development. The results reveal variations in cytokine production and expression of MHC, as well as costimulatory and inhibitory molecules in T- and B-lymphocytes between both mouse models that consistently result in differences in their ability to modulate T-lymphocyte responses. The results also highlight the capacity of B-lymphocytes to shape T-lymphocyte phenotypes and show that T- and B-lymphocytes of both transgenic mice exert disparate effect or functions, thus indicating why the development of the disease in both mice models is so different.

## Materials and Methods

### Mice

NOD, NOD-*PerIg*, and 116C-NOD mice were maintained by brother-sister mating under specific pathogen-free conditions at the University of Lleida. This study was carried out in accordance with the principles of the Basel Declaration and recommendations of the Catalan Government (*Generalitat de Catalunya*) concerning the protection of animals for experimentation. The protocol was approved by the Committee on the Ethics of Research in Animal Experimentation of the University of Lleida, Spain. Protocol #: CEEA 02-04/16.

### Diabetes Incidence

Mice were checked weekly for glycosuria with Glucocard strips (Meranini, Barcelona, Spain). After two positive readings (>3+) and with a confirmation of glucose levels on blood (higher values than 250 mg/dL), mice were considered diabetic.

### Flow Cytometry

Splenocytes and islet-infiltrating leukocytes from 6-week-old female NOD, 116C-NOD and NOD-*PerIg* mice were obtained as described elsewhere (16) and then analyzed by flow cytometry using FACS CANTO II instrumentation (BD, Biosciences, San Jose, CA) and Flowjo (version 8.7) software [see also Supplementary Figure 2 for histogram analysis for fluorescence minus one (FMO) control of each staining and for a representative analysis of transcription factor expression in CD4+ T lymphocytes]. CD19-V450 (75-0193-U100) was obtained from Tonbo Biosciences (San Diego, CA). CD3-FITC (561798), CD40-Biotin (553789), CD8-Biotin (553033), CD4-PerCp (553052), CD279-APC (562584), CD274-PacificBlue (564715), CD86-PE (561963), CD80-Biotin (09602D), I-Ak-Biotin (553539) which cross-reacts with I-Ag7, H-2K^d^-Biotin (553564), Fc(CD16/CD32)-PeCy7 (560829), and streptavidin-amCyan (563261) were purchased from BD Biosciences (San Jose, CA). Fas-APC (152603) was supplied by Biolegend (San Diego, CA). TACI-Biotin (ABIN1169259) and BAFF-R-Biotin (ABIN1169050) were purchased from Antibodies-online.com (USA). BAFF-APC (130-111-029) was obtained from Miltenyi Biotec (Madrid, Spain). For transcription factors and APRIL intracellular staining, mononuclear cells were permeabilized following the manufacturer's instructions of the FoxP3 Stainning Buffer Set (00-5523-00, ebioscience) and using the following set of MoAbs to T-bet-Pe/Cy7 (25-5825-82), RORgt-APC (53-9966-42), FoxP3-PacificBlue (48-5773-82), GATA3-FITC (53-9966-42), all from ebioscience (San Diego, CA), and APRIL-APC (130-105-388) from Miltenyi Biotec (Madrid, Spain).

### Proliferation Assays

Splenocytes from 6-week-old female NOD, BDC2.5-NOD, 116C-NOD, and NOD-*PerIg* mice were obtained mechanically disrupting their spleens, and red cells were lysed in 0.87% ammonium chloride solution. Spleens from BDC2.5-NOD mice were donated by Dr. Pau Serra from his mouse colony at the Institut d'Investigacions Biomèdiques August Pi i Sunyer, Barcelona, Spain. T- and B-lymphocytes were purified by negative selection using MACS T- and B-lymphocyte isolation reagent (130-095-130 and 130-090-862 respectively, Myletnyi Biotec, Madrid, Spain) following the manufacturer's instructions. The purity of T- and B-lymphocytes was always above 90%. *For B proliferation assays*, previous CFSE (C34554, life technologies) labeled B-lymphocytes were incubated in 96-well tissue culture plates (3 × 10^5^/well) at 37°C in 5% CO_2_ in complete culture medium using the following stimuli: 10 ug/ml LPS (L3012-5MG, Sigma-Aldrich Inc., St. Louis, MO), or 10 ug/mL anti-CD40 (553787, BD Biosciences, clone 3/23) plus 10 U/mL rIL-4 (404-ML-010, R&D Systems, Minneapolis, MN), or anti-IgM, affinity pure F(ab′)2 (JAC-715-006-020, Jackson Immunoresearch, West Grove, PA). After 48 h of culture, the phenotype of B-lymphocytes was analyzed by flow cytometry and culture supernatants were stored at −80°C until use. A minimum of four animals per group was analyzed. *For T-lymphocyte proliferation assays*, previous CFSE labeled T-lymphocytes from spleens of NOD or BDC2.5-NOD mice were co-cultured (3 × 10^5^ cell/well) with purified B-lymphocytes (3 × 10^5^ cell/well) of NOD, NOD-*PerIg* or 116C-NOD to check B-lymphocyte capacity to promote T-lymphocyte proliferation. 10 ug/mL of anti-CD3 MoAb (553058, BD), or 20 ug/mL of 2.5HIP peptide (LQTLALWSRMD) synthesized by the proteomics and protein chemistry unit of Pompeu Fabra University (Barcelona), was also added to cultures or anti-CD3 coated plates to boost T-lymphocyte activation. Seventy-two hours later, cells were analyzed by flow cytometry and culture supernatants stored at −80°C until use. All B- and T-lymphocyte proliferation assays were performed in duplicate.

### Cytokine Profile Analysis

Culture supernatants from proliferation assays were collected and IL-2, IL-4, IL-6, IFN-γ, IL-10, TNF-α, and IL-17 cytokine amounts analyzed by flow cytometry using CBA kit (560485, BD).

### Statistics

PRISM Graphpad software was used for analysis. Statistics were performed using log-rank (Mantel-Cox) and Mann-Whitney *U-*tests. All mean values ± SD are shown.

## Results

### Marked Phenotype Differences Between B-Lymphocytes From 116C-NOD and NOD-*PerIg* Mice

**Figure 1:**
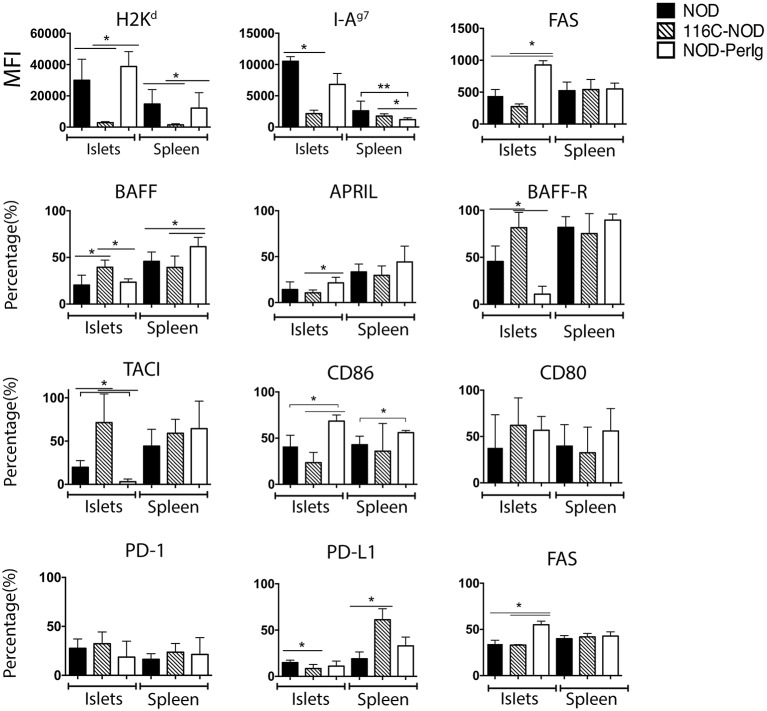
Expression of MHC, costimulatory and coinhibitory molecules in B-lymphocyte membrane. Median fluorescent intensity (MFI) of H2K^d^, I-Ag^7^, and FAS expression, and percentage values of BAFF, APRIL, BAFF-R, TACI, CD86, CD80, PD-1, PD-L1, and FAS positive islet-infiltrating or splenic B-lymphocytes from 6-week-old NOD, 116C-NOD and NOD-*PerIg* were analyzed (*N* = 5 per group, with two replicates for each experimental condition). Bars show the SD of the median of MFI or from the percentage values. Mann-Whitney U was used for analysis and statistically significant differences are shown as *P < 0.05 or **P < 0.01.

Since B-lymphocytes play an important role in presenting ß-cell autoantigens to autoreactive T-lymphocytes in autoimmune diabetes, the present study first analyzed the expression of MHC and CD80, CD86, BAFF, BAFF-R, APRIL, and TACI costimulatory and coinhibitory molecules in spleen and islet infiltrating B-lymphocytes (IIBLs) from 116C-NOD and NOD-*PerIg* mice. MHC class I and class II expression were deeply down-regulated in both IIBLs and splenic B cells from 116C-NOD, whereas only MHC class II was down-regulated in splenic B cells from NOD-*PerIg* mice compared to wild type NOD mice (Figure 1). In regard to costimulatory molecules, the percentages of IIBLs positive for CD86 and APRIL were lower, but higher for BAFF, BAFF-R, and TACI in 116C-NOD when compared to wild type NOD and/or NOD-*PerIg* mice (Figure 1). In fact, the number of IIBLs positive for BAFF-R and TACI was almost absent in NOD-*PerIg*. Since TACI acts as an inhibitor for lymphocyte activation through BAFF/BAFF-R interaction, these results suggest that 116C-NOD IIBLs cannot induce activation signals for T cells. In contrast, the high percentage of IIBLs with up-regulation of costimulatory molecules suggests a high capacity of these cells from NOD-*PerIg* mice to be potential T-lymphocyte activators. The level and number of Fas+ IIBLs was higher in NOD-*PerIg* compared to NOD and 116C-NOD mice, thus suggesting that IIBLs from NOD-*PerIg* mice exhibit a high activation status. An increased percentage of BAFF+ B-lymphocytes is observed in the spleen of NOD-*PerIg* when compared to 116C-NOD and wild type NOD mice. A number of other less pronounced differences in the percentage or the expression of above mentioned molecules in splenic B-lymphocytes were also observed between mouse strains (Figure 1). The expression of PD-1 and PD-L1 coinhibitory molecules was then tested in IIBLs and splenic B-lymphocytes from 116C-NOD and NOD-*PerIg* mice. No differences were observed in the percentage of PD-1+ B-lymphocytes between mice. On the contrary, the percentage of PD-L1+ in IIBLs was lower in all three mouse models (Figure 1) suggesting low ability of B-lymphocytes to down regulate T-lymphocyte activity in islet pancreatic-infiltrate.

**Figure 2:**
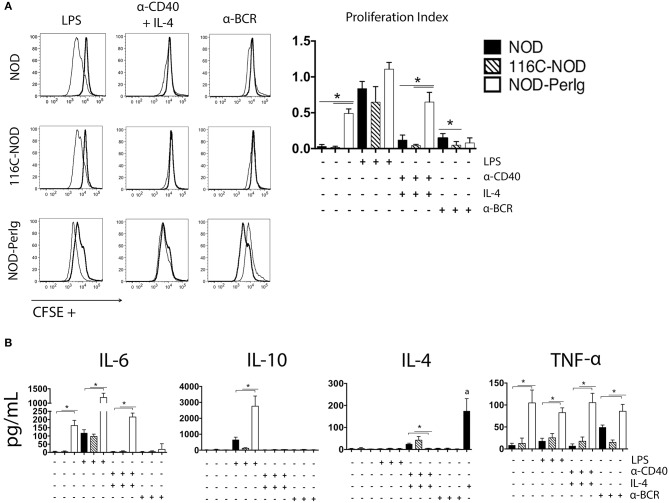
B-lymphocyte proliferation assay. **(A)** CFSE analysis of proliferation responses of splenic B-lymphocytes from 6-week-old female NOD, 116C-NOD and NOD-*PerIg* mice (*n* = 4 per group, with two replicates for each experimental condition). Splenic B-lymphocytes were purified, labeled with CFSE and cultured in the presence (narrow line) of LPS, anti-CD40 + IL-4, anti-BCR stimuli or without stimulus (bold line) for 48 h. **(B)** IL-4, IL-6, TNF-α, and IL-10 cytokine concentrations were assessed in culture supernatants of purified B lymphocytes cultured in the same conditions as described above. (a) = Amount of IL-4 added to the culture media in absence of cells. Bars show the SD of the proliferation index or from the cytokine concentration values. Mann-Whitney U was used for analysis and statistically significant differences are shown as *P < 0.05.

In addition to the phenotypic changes, the ability to respond to different stimuli determines the functional capacity of B-lymphocytes. As previously demonstrated, 116C B-lymphocytes have a decreased ability to proliferate but can still secrete some cytokines in response to LPS, anti-CD40 + IL-4, and anti-BCR (13). Proliferation and cytokine secretion analysis under the same stimuli were carried out with splenic B-lymphocytes from NOD-*PerIg*, 116C-NOD, and wild type NOD mice to compare their activation capacity. Significant differences were observed in the proliferation assays (Figure 2A). Interestingly, compared to 116C-NOD and wild type NOD mice, NOD-*PerIg* B-lymphocyte proliferation was higher at baseline and under anti-CD40 + IL-4 stimuli. Of note, under anti-BCR stimulation, NOD-*PerIg* B-lymphocytes did not proliferate (Figure 2A), and an increased number of dead cells was observed in the SSC-A/FSC-H flow cytometry analysis compared to baseline conditions (Supplementary Figure 3). Since NOD-*PerIg* B-lymphocytes seem to be already highly activated just after isolation (baseline condition), these results suggest that they might have undergone activation-induced cell death after BCR stimulation. Significant differences were also observed in the B-lymphocyte cytokine production of the different mouse strains. B-lymphocyte secretion of TNF-α to all different stimuli, IL-6 secretion after LPS and anti-CD40 + IL-4, and IL-10 secretion after LPS stimulation was significantly higher in NOD-*PerIg* compared to 116C-NOD and wild type NOD mice (Figure 2B). Interestingly, B-lymphocytes from NOD-*PerIg* mice secreted high amounts of IL-6 and TNF-α cytokines, even without stimulation. Despite the external supply of IL-4, almost no IL-4 was detected in the B-lymphocyte culture wells under the stimulus of anti-CD40 + IL-4, thus suggesting a high uptake of this cytokine by NOD-*PerIg* B-lymphocytes during the culture period compared to 116C-NOD and wild type NOD mice (Figure 2B). Altogether, these results suggest that NOD-*PerIg* B-lymphocytes display an activated phenotype and, consequently, might be able to activate autoreactive T-lymphocytes and induce autoimmune diabetes development more efficiently than 116C-NOD B-lymphocytes.

### Spleen and Islet Infiltrating T-Lymphocytes From 116C-NOD and NOD-*PerIg* Mice Are Phenotypically Different

**Figure 3:**
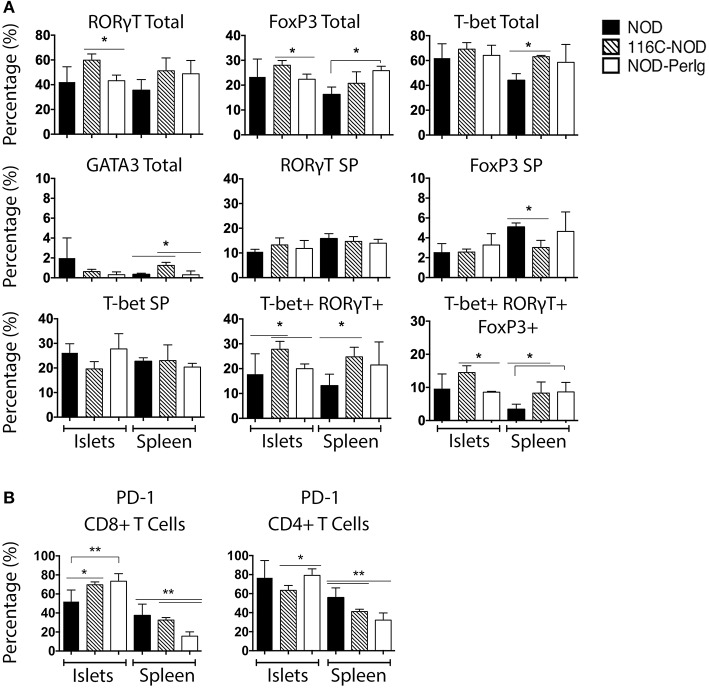
Phenotype of unstimulated splenic and islet infiltrating NOD, 116C-NOD, and NOD-*PerIg* T-lymphocytes. **(A)** T-bet, RORγt, GATA3, and FoxP3 expression was assessed by flow cytometry in splenic and islet-infiltrating T-lymphocytes from 6-week-old female NOD, 116C-NOD and NOD-*PerIg* mice (*n* = 4 per group, with two replicates for each experimental condition). “Total” and “SP” stand for the percentage of “whole positive” and “single positive” cells for the aforementioned marker, respectively. **(B)** Expression of PD-1 was analyzed by flow cytometry in the spleen and islet-infiltrating T-lymphocytes from 6-week-old female NOD, 116C-NOD and NOD-*PerIg* mice (*n* = 4 per group, with two replicates for each experimental condition). Bars show the SD of the percentage values. Mann-Whitney U, statistically significant differences: *P < 0.05 or **P < 0.01.

Since the functional status of B-lymphocytes can decisively impact T-lymphocyte behavior, the phenotypic characteristics of spleen and islet-infiltrating T-lymphocytes were analyzed in NOD, 116C-NOD, and NOD-*PerIg* mice. As previously reported (12), B-lymphocytes from 116C-NOD mice induced a shift toward a dominant T-lymphocyte Th17 differentiation, especially through double Th1/Th17 bias compared to NOD mice (Figure 3A). However, no significant differences were found regarding the T helper phenotype from CD4+ T-lymphocytes between NOD-*PerIg* and NOD mice either in the spleen or in the pancreatic islet infiltrates. Only a significant increase in triple T-bet+RORγt+FoxP3+ CD4+ T-lymphocytes was detected in the spleen of NOD-*PerIg* compared to NOD mice. The expression of PD-1 and PD-L1 by splenic and islet infiltrating CD4+ and CD8+ T-lymphocytes from NOD, 116-NOD and NOD-*PerIg* mice was then analyzed (Figure 3B). A significant increase in the expression of PD-1 was observed in CD4+ IITLs from NOD-*PerIg* compared to 116C-NOD, and in CD8+ IITLs from NOD-*PerIg* and 116C-NOD compared to NOD mice. These data indicate that the CD8+ IITLs in both NOD-*PerIg* and 116C-NOD mice have a highly activated phenotype. However, a high percentage of CD4+ IITLs express PD-1 only in NOD-*PerIg*, suggesting that full activation of the IILTs occurs just in these mice.

### NOD-*PerIg* B-Lymphocytes Induce High CD4+ and CD8+ T-Cell Activation

**Figure 4:**
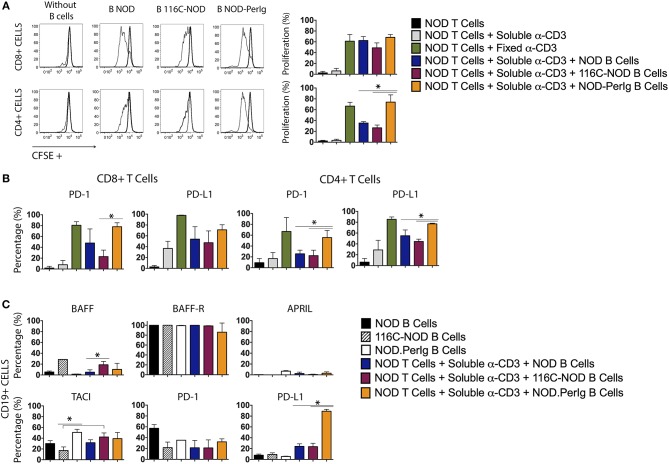
Proliferation assay and molecular marker expression by NOD T-lymphocytes co-cultured with B-lymphocytes from NOD, 116C-NOD, and NOD-*PerIg* mice. **(A)** NOD T-lymphocyte proliferation assay. Purified splenic T-lymphocytes from NOD mice were CFSE-labeled and cultured in the presence (narrow line) of soluble anti-CD3, or plate-coated anti-CD3, and either purified NOD, 116C-NOD, or NOD-*PerIg* B-lymphocytes plus anti-CD3, or without stimulus (bold line) for 72 h. Ten NOD mice were used to obtain the T-lymphocyte pool and 4 mice of each group to obtain purified B-lymphocytes. Two replicates were carried out for each experimental condition. The proliferation of CD4+ or CD8+ T-lymphocytes was analyzed independently. **(B)** The expression of PD-1 and PD-L1 in CD4+ and CD8+ NOD T-lymphocytes was analyzed after co-culturing them as previously described. **(C)** The proportion of NOD, 116C-NOD, and NOD-*PerIg* B-lymphocytes expressing BAFF, APRIL, BAFF-R, TACI, PD-1, or PD-L1 was also assessed. All B-lymphocytes were incubated alone without stimulus or in the presence of NOD T-lymphocytes plus anti-CD3 for 72 h (*n* = 4 per group, with two replicates for each experimental condition). Bars show the SD of the percentage values. Mann-Whitney U, statistically significant differences: *P < 0.05.

A growing body of evidence indicates that B-lymphocytes can contribute to autoimmune diabetes development by acting as APCs activating ß-cell autoreactive T-cells (1, 4, 10, 17–20). However, it has also been demonstrated that some B-lymphocytes can protect from disease development by inducing ß-cell autoreactive CTLs apoptosis or/and inhibiting APC activity (11). Previous studies with 116C-NOD and NOD-*PerIg* mice support the idea that B-lymphocytes can perform both actions depending on their functional status (12, 13). The present study indicates that the profound phenotypic and functional differences between 116C-NOD and NOD-*PerIg* B-lymphocytes could explain their dual role in respectively inhibiting or promoting autoimmune diabetes development. Thus, the ability of B-lymphocytes from 116C-NOD and NOD-*PerIg* to induce distinct NOD T-cell responses was then evaluated. The ability of 116C-NOD and NOD-*PerIg* B-lymphocytes to induce T-lymphocyte proliferation was analyzed first. The results showed that NOD-*PerIg* B-lymphocytes had a higher capacity than those from 116C-NOD and NOD mice to induce CD4+ T-lymphocyte proliferation (Figure 4A). Interestingly, the percentage of PD-1 positive CD4+ and CD8+ T-lymphocytes was significantly higher when co-cultured with NOD-*PerIg* B-lymphocytes compared to those co-cultured with B-lymphocytes from NOD and/or 116C-NOD mice (Figure 4B), thus confirming the T-lymphocyte activation status. Next, the expression of BAFF, BAFF-R, APRIL, and TACI costimulatory molecules, and PD-1 and PD-L1 was also assessed in B-lymphocytes after co-culture with T-cells. The results indicated that a significant number of B-lymphocytes from NOD-*PerIg*, but not from NOD or 116-NOD mice, expressed PD-L1, thus being protected from the CTLs killing (Figure 4C). These results indicated that NOD-*PerIg* B-lymphocytes display a highly activated phenotype that promoted both CD4+ and CD8+ T-lymphocyte activation.

### B-Lymphocytes From NOD-*PerIg* Mice Enforce NOD T Cells Toward a Quadruple T-Bet+ GATA3+ RORγT+ Foxp3+ Phenotype

**Figure 5:**
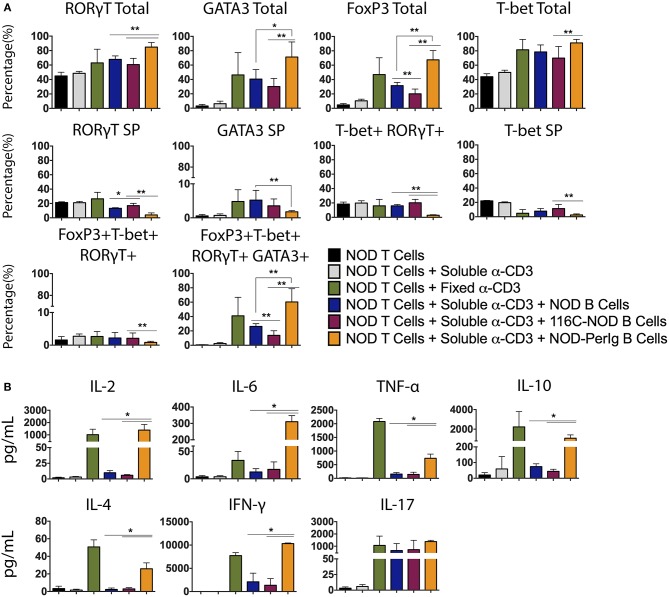
Phenotype analysis of NOD T-lymphocytes after culture with B-lymphocytes from NOD, 116C-NOD, and NOD-*PerIg* mice. **(A)** Transcription factors expression analysis in splenic NOD T-lymphocytes cultured in the presence of soluble anti-CD3, or plate-coated anti-CD3, or either purified NOD, 116C-NOD, or NOD-*PerIg* B-lymphocytes plus anti-CD3, or without stimulus for 72 h (*n* = 6, with two replicates for each experimental condition). “Total” and “SP” stand for the percentage of “whole positive” and “single positive” cells for the aforementioned marker, respectively. **(B)** Cytokine concentration analysis in culture supernatants of NOD T-lymphocytes cultured in the same condition as described above. Bars show the SD of the percentage values or cytokine concentrations (*n* = 4, with two replicates for each experimental condition). Mann-Whitney U, statistically significant differences: *P < 0.05 or **P < 0.01.

As previously shown, B-lymphocytes from 116C-NOD mice induce a shift toward a dominant T-lymphocyte Th1/Th17 differentiation (12). In the present study, the capacity of B-lymphocytes from NOD-*PerIg* mice to modulate and conduct CD4 T helper responses was evaluated. As in previous assays, transcription factor expression and cytokine production by T-lymphocytes from NOD mice after co-culture with B-lymphocytes from NOD-*PerIg*, 116C-NOD or wild type NOD mice was analyzed. Interestingly, the results indicated that B-lymphocytes from NOD-*PerIg* mice led most NOD CD4+ T-lymphocytes toward a quadruple T-Bet+ GATA3+ RORγT+ Foxp3+ phenotype (Figure 5A). Moreover, cytokine production analysis showed that B-lymphocytes from NOD-*PerIg* mice prompted NOD T-lymphocytes to secrete significant amounts of cytokines from different T helper subsets, although with predominance of proinflammatory and Th1 cytokines, due to large amounts of IFN-γ secretion (Figure 5B). These results indicate that B-lymphocytes from NOD-*PerIg* mice are highly active that mainly promote Th1 responses but not exclusively, confirming that B-lymphocytes can enforce CD4 T-lymphocytes toward different T helper functions depending on their phenotype and behavior.

### B-Lymphocytes From NOD*-PerIg* Presenting the 2.5HIP Epitope Promote High BDC2.5-NOD T-Lymphocyte Activation

**Figure 6:**
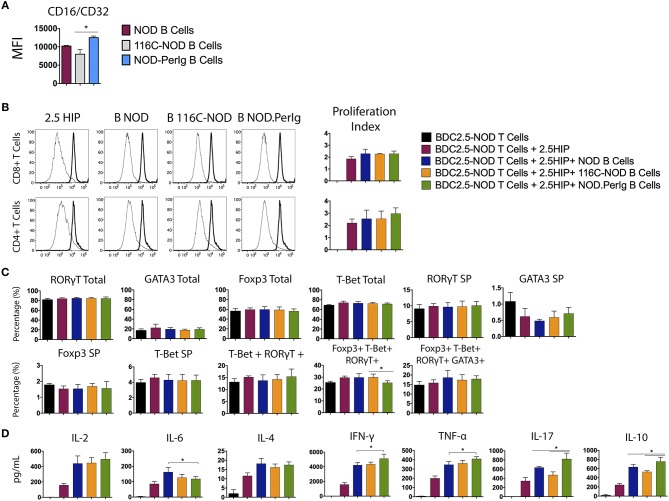
Analysis of BDC2.5-NOD T-lymphocyte activation after co-culture with B-lymphocytes from NOD, 116C-NOD, and NOD*-PerIg* mice. **(A)** The expression of Fc Receptors (CD16/CD32) from B-lymphocytes was analyzed by flow cytometry before culture (*n* = 4 per group). **(B)** BDC2.5**-**NOD T-lymphocyte proliferation assay. Purified splenic T-lymphocytes from BDC2.5-NOD mice were CFSE-labeled and cultured in the presence of 2.5HIP alone and with purified NOD, 116C-NOD, or NOD-*PerIg* B-lymphocytes, or without stimulus for 72 h. **(C)** Transcription factor expression was analyzed after culturing cells as mentioned above. “Total” and “SP” stand for the percentage of “whole positive” and “single positive” cells for the aforementioned marker, respectively. **(D)** Cytokine release was checked in supernatants after 72 h. Bars show the SD of the percentage values or cytokine concentrations. In all experiments *N* = 4. Mann-Whitney U, statistically significant differences: *P < 0.05.

In the above experiments, the differences observed in T-lymphocyte activation could be due to the ability of anti-CD3 antibody to bind to B-lymphocytes of the different mouse strains. To shed light on this issue, Fc receptor (CD16/CD32) expression on NOD*-PerIg*, NOD, and 116C-NOD B-lymphocytes was analyzed. Higher Fc expression was observed in NOD*-PerIg* compared to NOD and 116C-NOD B-lymphocytes (Figure 6A). Thus, in order to confirm that the ability of NOD*-PerIg* B-lymphocytes to activate T-lymphocytes was due to their high capacity to express co-stimulation molecules and cytokine secretion, rather than or only by the increase in anti-CD3 binding capacity, BDC2.5-NOD T-lymphocyte activation assays were performed. To this end, T-lymphocytes from BDC2.5-NOD were incubated with their 2.5HIP cognate epitope in the presence of B-lymphocytes from NOD, 116C-NOD, and NOD*-PerIg* mice. Although the proliferation of T-lymphocytes was not as high as expected, NOD*-PerIg* B-lymphocytes were able to induce more efficiently CD4 T-lymphocyte proliferation than their counterparts (Figure 6B). Although no differences in the transcription factor expression were detected (Figure 6C), BDC2.5-NOD T-lymphocytes co-cultured with NOD*-PerIg* produced larger amounts of cytokines than with NOD or 116C-NOD B-lymphocytes (Figure 6D). In fact, BDC2.5-NOD T-lymphocytes produced larger amounts of different cytokines distinctive of different T-lymphocyte subsets. Surprisingly, BDC2.5-NOD T-lymphocytes incubated with 116C-NOD proliferate as efficiently as when they were in the presence of B-lymphocytes from NOD. These results suggest the ability of 116C-NOD B-lymphocytes to overcome their “anergic” state in order to stimulate T-lymphocytes in the presence of an autoantigen. Nevertheless, their ability to induce cytokine production was not as good as the one of NOD or NOD*-PerIg* B-lymphocytes.

## Discussion

It is currently well accepted that B-lymphocytes contribute to the development of autoimmune diabetes. The lack of B-lymphocytes in NOD mice prevents disease development (1–8), whereas depending on their antigenic specificity the transgenic expression of BCRs either induces, delays, or prevents autoimmune diabetes (10, 12, 13, 21–24). The diabetogenic capacity of B-lymphocytes in two BCR transgenic mice, the NOD-*PerIg* and the 116C-NOD strains (12, 13) was analyzed in previous studies. In 116C-NOD mice, B-lymphocytes display an anergic-like phenotype delaying autoimmune diabetes onset and decreasing disease incidence, whereas in NOD-*PerIg* mice, B-lymphocytes acquire an activated proliferative phenotype that promotes disease development. Moreover, a T helper response imbalance toward a Th17 phenotype was observed in 116C-NOD compared to NOD mice, thus suggesting a strong linkage between B-lymphocyte phenotype and T-lymphocyte response.

By comparing the phenotype of the three mouse strains, the NOD, the 116C-NOD, and the NOD-*PerIg*, the present study sought to determine the most relevant B-lymphocyte traits during autoimmune diabetes. To this end, the linkage between phenotypic characteristics of B-lymphocytes and the ability of these cells to induce phenotypic and functional changes in T-lymphocytes was further analyzed. To this end, the expression of accessory molecules and cytokine secretion in B-lymphocytes from NOD-*PerIg*, 116C-NOD, and NOD mice was evaluated. Then, through a nonspecific anti-CD3 stimulus that mimics a signal through the TCR, the ability of these B-lymphocytes to induce T-lymphocyte activation was also analyzed. With this approach, the effect was assessed independently from their antigenic specificity of expressed accessory molecules and secreted cytokines by the B-lymphocytes in the activation of the T-lymphocytes. Finally, B lymphocyte capacity to act as APCs in autoimmune diabetes environment was tested by analyzing the ability of these cells to present a known diabetes-related autoantigen, known as 2.5HIP, a Hybrid Insulin Peptide. In contrast to those from the NOD and 116C-NOD strains, B-lymphocytes from NOD-*PerIg* mice display an activated phenotype, secrete high amounts of proinflammatory cytokines (even without any B-lymphocyte stimulation), and induce significant T-lymphocyte activation. In addition, B-lymphocytes from116C-NOD express significantly lower levels of MHC molecules compared to those from NOD and NOD-*PerIg* mice, thus indicating their inability to perform a good antigen presentation. Although a previous study showed that CD86 expression levels in B-lymphocytes from 116C-NOD mice were high (12) suggesting perhaps a greater capacity for activation of T-lymphocytes, the percentage of B-lymphocytes expressing CD86 is lower in 116C-NOD than in NOD and NOD-*PerIg* mice. Moreover, since BAFF-R and TACI is higher in 116C-NOD than in NOD and NOD-*PerIg* mice and taking into account that TACI acts as an inhibitor for lymphocyte activation through BAFF/BAFF-R interaction, these results suggest that 116C-NOD IIBLs cannot induce a strong T-lymphocyte activation. In fact, current and previous data (12) suggest that 116C-NOD B-lymphocytes would show a greater tendency to fostering a response toward Th17 phenotype. In contrast to the 116C-NOD B-lymphocytes, the expression levels of MHC molecules in B-lymphocytes from NOD-*PerIg* mice are similar to those from NOD mice. In addition, the percentage of B-lymphocytes expressing high levels of the CD86 molecule and low levels of TACI and BAFF-R is higher in NOD-*PerIg* mice compared to NOD and 116C-NOD mice, indicating the strong ability of B lymphocytes to activate T lymphocytes. Consistent with these results and those obtained in the analysis of the incidence of autoimmune diabetes, this study also shows that the NOD-*PerIg* B-lymphocytes have the capacity to proliferate and secrete proinflammatory cytokines, even in the absence of any stimulus, and induce a strong T-lymphocyte proliferation. Moreover, the results also indicate that NOD-*PerIg* B-lymphocytes induce CD4+ T-lymphocytes to differentiate toward a quadruple Bet+ GATA3+ RORγT+ Foxp3+ phenotype and to secrete cytokines characteristic from most different T helper subsets, although with predominance of proinflammatory and Th1 cytokines, thus contributing to autoimmune diabetes development (25). These findings were confirmed when we analyzed the ability of the different B-lymphocytes to induce specific responses to the known diabetes-related peptide 2.5HIP. Even without a polyclonal stimulus, NOD*-PerIg* B-lymphocytes were able to activate specific responses from BDC2.5-NOD T-lymphocytes, inducing a higher production of different cytokines when compared to the other two strains. On the contrary, no changes were found between strains in the phenotype of BDC2.5-NOD CD4+ T-lymphocytes after stimulation with the 2.5HIP cognate epitope. This could be due to their transgenic nature, being their prefixed phenotype strong enough to support any input, compared to NOD CD4+ T-lymphocytes. As observed in previous works carried out with the transgenic model 125tg-NOD (20), our results indicate that 116C-NOD B-lymphocytes can induce T-lymphocyte activation and cytokine production in very specific situations despite their “anergic phenotype.”

Thus, this study also clearly shows that depending on their phenotype and behavior (secreted cytokine and accessory molecule expression), B-lymphocytes from NOD, 116C-NOD, and NOD-*PerIg* mice can promote CD4+ T lymphocytes toward different T helper functions. The present findings also illustrate that to face any insult, the immune system can bring a combined T helper response exerted by several CD4+ T-lymphocyte populations if required. Moreover, since the immune system has an enormous plasticity and redundancy, other CD4+ T-lymphocytes populations of T helper that are phenotypically double, triple or even quadruple Th positive may also be found (26). For instance, CD4+ T-lymphocytes that are at the same time Th1 and Th17, or other multiple possible combinations, even some that simultaneously display multiple Th type markers. A cell population can also pass from one phenotype to another in the presence of certain cytokines. For example, a subpopulation of Th9 can be differentiated from Th2 in the presence of TGF-β (27).

In summary, this study highlights the existing interdependence between B- and T-lymphocytes, which gives to the former populations the chance to set up T-lymphocyte phenotypes through the secretion of cytokines and the expression of costimulatory and co-inhibitory molecules. In terms of autoimmune diabetes, this study also confirms that the most harmful response for disease development comes from a concomitant Th1/proinflammatory response, and that B-lymphocytes play the key role in the establishment of this response.

## Ethics Statement

This study was carried out in accordance with the recommendations of Catalan and Spanish Government guidelines for Animal Experimentation (Directiva 2010/63/UE; Real Decreto 53/2013; Llei 5/1995/GC; Orden 214/1997/GC). Ethical Committee of Animal Experimentation of the University of Lleida. Experimental procedure # CEEA, 02-04/16.

## Author Contributions

LE-M researched data and wrote the manuscript. BA, ER-M, MC-P, JCarrascal, JCarrillo, HC, and AP researched data. CM, MV-P, and TS contributed to discussion, and reviewed the manuscript. DS co-conceived the study, contributed to discussion, and reviewed manuscript. JV conceived the study, evaluated data, wrote, reviewed and edited to the manuscript. JV is the guarantor of this work and, as such, had full access to all the data in the study and takes responsibility for the integrity of the data and the accuracy of the data analysis. All authors revised the work and gave final approval of the version to be published.

### Conflict of Interest Statement

The authors declare that the research was conducted in the absence of any commercial or financial relationships that could be construed as a potential conflict of interest.
