# Supplementary figures and images for: B-Lymphocyte Phenotype Determines T-Lymphocyte Subset Differentiation in Autoimmune Diabetes
Source: Front Immunol. 2019 Jul 25;10:1732. doi: 10.3389/fimmu.2019.01732 (PMC6689997; doi:10.3389/fimmu.2019.01732)

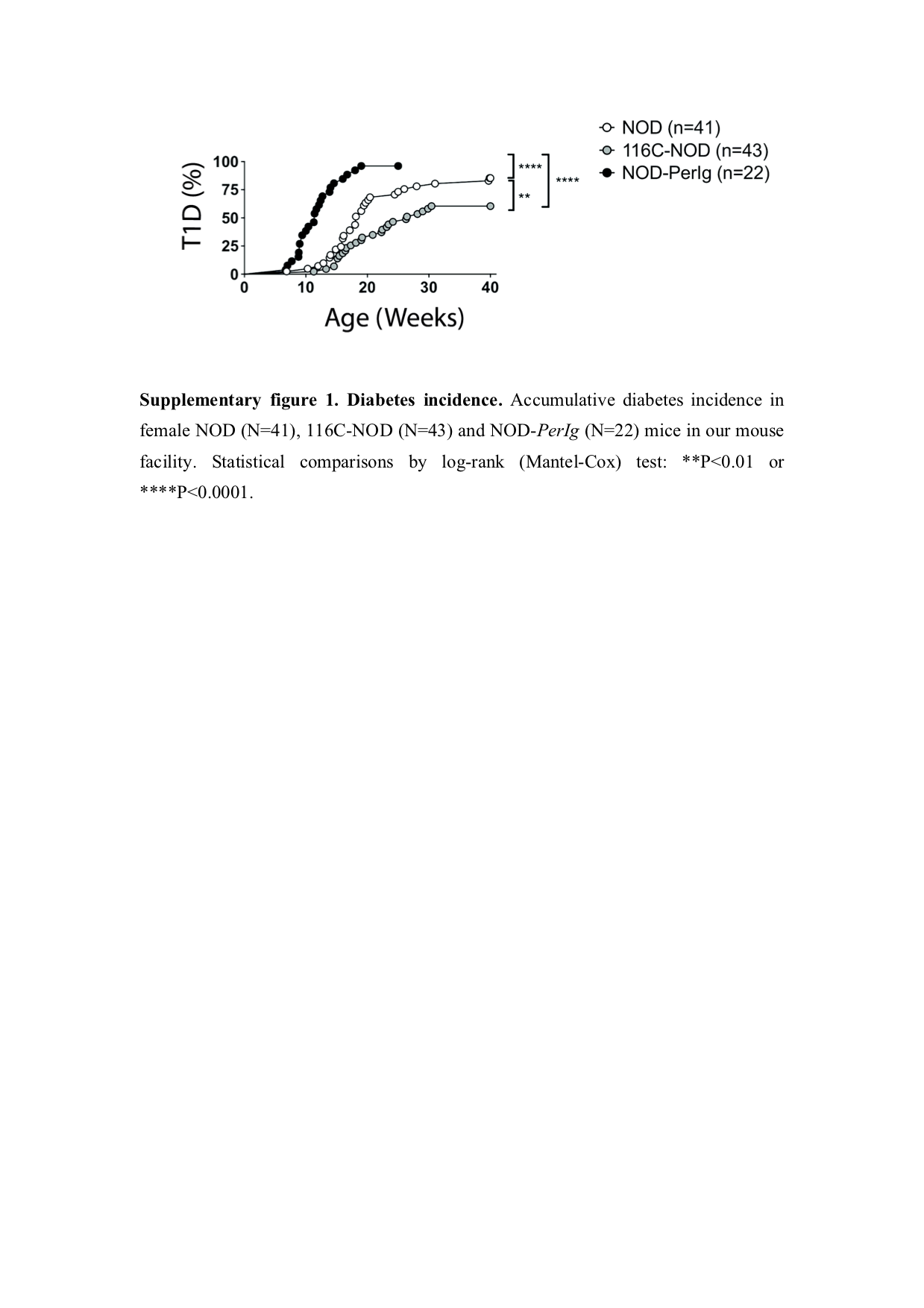

Supplement: Supplementary file 1 [file Image_1.TIFF]

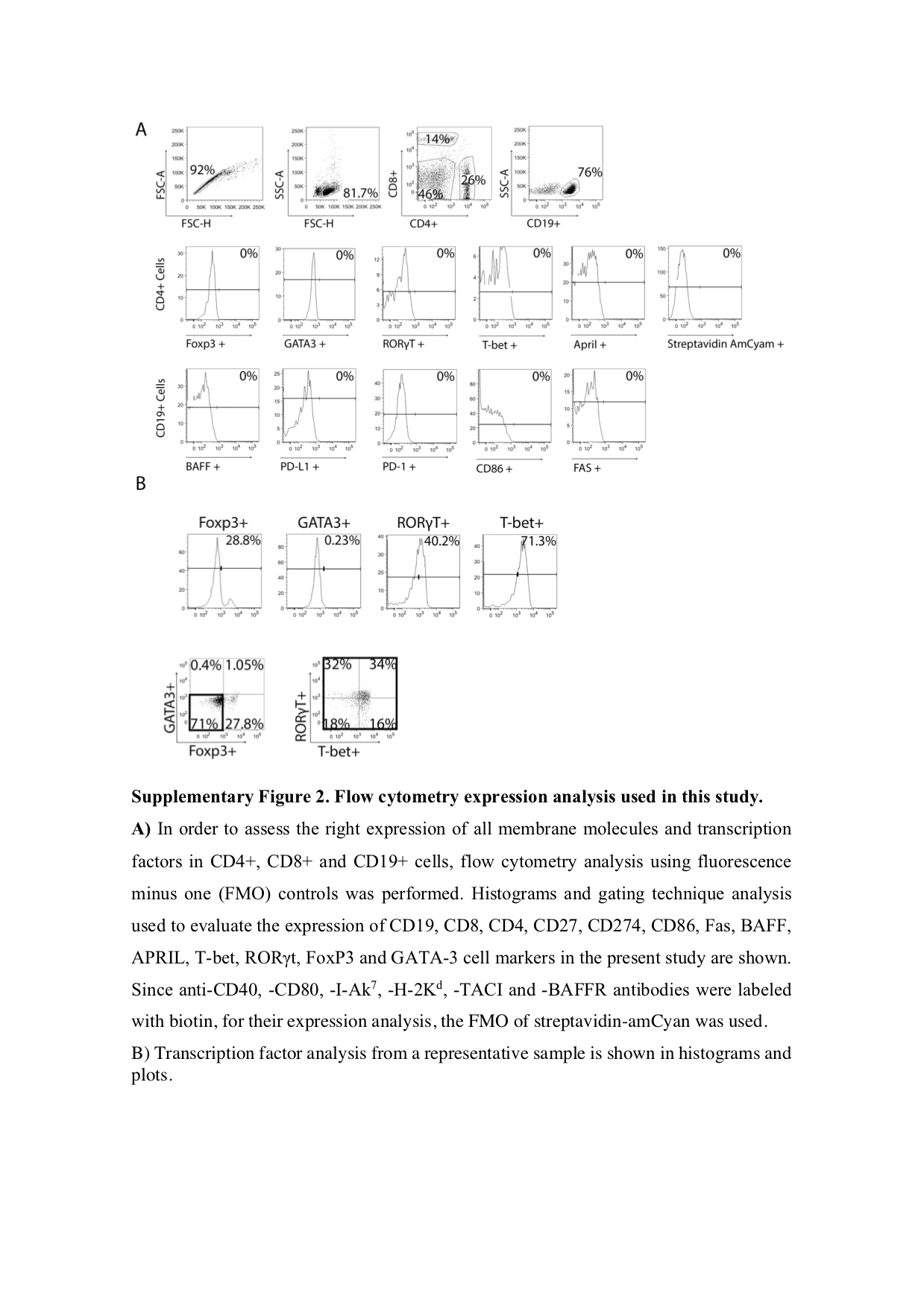

Supplement: Supplementary file 2 [file Image_2.tiff]

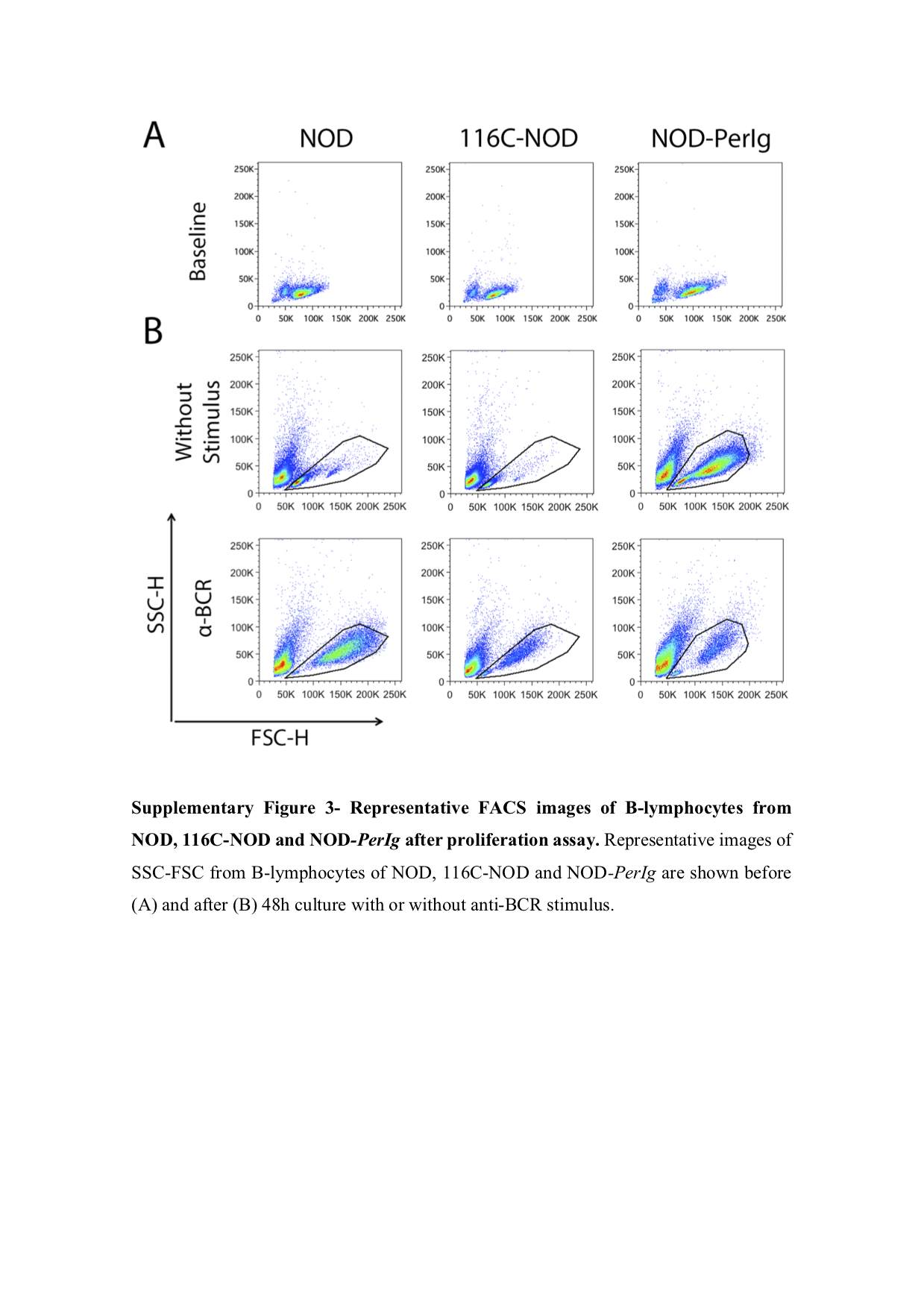

Supplement: Supplementary file 3 [file Image_3.tiff]
